# Epigenetics in Skin Homeostasis and Ageing

**DOI:** 10.3390/epigenomes9010003

**Published:** 2025-01-09

**Authors:** Iasonas Dermitzakis, Stella Aikaterini Kyriakoudi, Sofia Chatzianagnosti, Despoina Chatzi, Efstratios Vakirlis, Soultana Meditskou, Maria Eleni Manthou, Paschalis Theotokis

**Affiliations:** 1 Department of Histology-Embryology, School of Medicine, Aristotle University of Thessaloniki, 54124 Thessaloniki, Greece; kstellaai@auth.gr (S.A.K.); sofiachatzianagnosti@gmail.com (S.C.); chatzidc@auth.gr (D.C.); sefthym@auth.gr (S.M.); mmanthou@auth.gr (M.E.M.); ptheotokis@auth.gr (P.T.); 2 First Department of Dermatology and Venereology, School of Medicine, Aristotle University of Thessaloniki, 54643 Thessaloniki, Greece; svakirlis@auth.gr

**Keywords:** epigenetics, DNA methylation, histone modification, non-coding RNAs, skin, epidermis, homeostasis, ageing

## Abstract

The skin, the largest organ of the human body, plays numerous essential roles, including protection against environmental hazards and the regulation of body temperature. The processes of skin homeostasis and ageing are complex and influenced by many factors, with epigenetic mechanisms being particularly significant. Epigenetics refers to the regulation of gene expression without altering the underlying DNA sequence. The dynamic nature of the skin, characterized by constant cellular turnover and responsiveness to environmental stimuli, requires precise gene activity control. This control is largely mediated by epigenetic modifications such as DNA methylation, histone modification, and regulation by non-coding RNAs. The present review endeavours to provide a comprehensive exploration and elucidation of the role of epigenetic mechanisms in regulating skin homeostasis and ageing. By integrating our current knowledge of epigenetic modifications with the latest advancements in dermatological research, we can gain a deeper comprehension of the complex regulatory networks that govern skin biology. Understanding these mechanisms also presents promising avenues for therapeutic interventions aimed at improving skin health and mitigating age-related skin conditions.

## 1. Introduction

The skin serves as the primary physical barrier protecting the body from the external environment. It is composed of three principal layers: the epidermis, dermis, and hypodermis [1,2]. The outermost layer, the epidermis, consists of keratinocytes and melanocytes, as well as Langerhans and Merkel cells [3]. This layer functions as a waterproof barrier, guarding against physical injury, ultraviolet (UV) damage, harmful chemicals, and infections [3,4,5]. The basement membrane separates the epidermis from the underlying dermis and also houses a population of epidermal stem cells (SCs). The dermis, the skin’s thickest layer, contains blood vessels, glands, hair follicles (HFs), and nerve endings [4,6,7]. This layer supplies nutrients to the epidermis and regulates body temperature [6,7]. Beneath the dermis is the innermost layer, the hypodermis, which comprises fibroblasts, adipocytes, and macrophages [6,7,8]. The hypodermis is an insulating layer that protects underlying muscles and organs and stores energy as fat [4,8]. The complex structure of the skin relies on tightly regulated cellular processes to maintain its protective functions and overall stability.

Skin homeostasis is a continuous process that begins during embryonic development and persists throughout life [2,9]. It involves balancing between SC proliferation and differentiation, a crucial process for maintaining tissue integrity and function [6,10,11]. Distinct populations of epidermal SCs are located in the interfollicular epidermis, HFs, infundibulum, and isthmus, as well as in the sebaceous and sweat glands [2,12]. Keratinocytes, which originate from interfollicular SCs, undergo several morphological and metabolic transformations, forming the distinct layers of the epidermis [13]. As these cells migrate from the basal layer to the surface of the epidermis, the types and numbers of cell junctions change, which enhances keratinocyte flexibility and tissue integrity [14]. However, the rate at which SC populations regenerate and produce daughter cells is influenced by external injuries, routine tissue turnover, and remodelling [15,16]. Similar to processes occurring in other tissues [17,18,19], the activity of skin SCs is meticulously regulated by an intricate network of molecular signals that sustain the equilibrium among quiescence, self-renewal, and differentiation [2,15].

Recently, the underlying molecular mechanisms of skin biology have been gradually elucidated. Epigenetic and metabolic regulations have emerged as key drivers of skin homeostasis, ensuring the proper balance between cell proliferation, differentiation, and tissue repair to preserve the integrity of the skin [10,20]. Additionally, epigenetic alterations are correlated with skin ageing, which influences cellular plasticity and governs cell function over time [10,21,22]. Epigenomics involves changes in gene expression without altering the DNA sequence and can be categorized into DNA methylation, histone tail modifications, and microRNA (miRNA) expression [20,23]. The disruption of these epigenetic processes can lead to various skin disorders, including psoriasis, atopic dermatitis, and skin cancer [24,25]. Recent advances in epigenetic biology suggest that epigenetic mechanisms represent promising therapeutic intervention targets, particularly in dermatology [26,27,28,29]. Unlike irreversible DNA alterations, epigenetic modifications are reversible, making them attractive candidates for drug development [30].

The present review aims to comprehensively explore and elucidate the role of epigenetic mechanisms in skin homeostasis and ageing. By synthesizing current research findings, the review seeks to highlight the critical influence of epigenetic regulation on cellular dynamics, tissue integrity, and the ageing process of the skin. Understanding epigenetic control is expected to foster the development of personalized, epigenetically targeted therapies, thereby enhancing treatment outcomes in dermatology. Such advancements could pave the way for more effective, individualized patient care by integrating epigenetic biomarkers into diagnostic and therapeutic strategies.

## 2. An Overview of Epigenetic Mechanisms

Epigenetics refers to modifications in gene expression that occur without changes to the underlying DNA sequence [31]. Key epigenetic changes include DNA methylation, hydroxymethylation, and post-translational histone modifications [20,32,33]. Additionally, miRNA-mediated gene regulation plays a significant role [24,33]. The combination of these modifications defines chromatin structure and influences gene accessibility for transcription [20,34]. In the current section, the epigenetic mechanisms of chromatin configuration and remodelling will be briefly described.

### 2.1. DNA Methylation

DNA methylation is the most extensively characterized epigenetic modification, involving the covalent addition of methyl groups (CH_3_) to specific genomic regions, particularly at transcriptionally active sites and gene promoters [35,36,37]. This modification predominantly occurs at cytosine–phosphate–guanine (CpG) dinucleotides, commonly referred to as CpG islands. These CpG islands comprise repetitive cytosine–guanine sequences, typically ranging from 0.5 to 4 kilobases in length, and are found in approximately 70–80% of the human genome [38,39]. Methylation involves transferring methyl groups to the cytosine ring’s 5-carbon position, resulting in transcriptional repression and gene silencing [36,40]. This biochemical process is mediated by a family of enzymes called DNA methyltransferases (DNMTs), which include DNMT1, DNMT3a, and DNMT3b. DNMT1 primarily functions in the maintenance of DNA methylation, sustaining methylation patterns during DNA replication in the S phase of the cell cycle by targeting hemimethylated DNA strands [41,42,43]. In contrast, DNMT3a and DNMT3b are classified as de novo methyltransferases, responsible for establishing new methylation marks in previously unmethylated genomic regions [35,42].

### 2.2. DNA Hydroxymethylation

DNA hydroxymethylation is an epigenetic mechanism that modifies DNA by adding a hydroxymethyl group to the 5-carbon position of cytosine, resulting in the formation of 5-hydroxymethylcytosine (5-hmC) [44,45]. This process is primarily catalyzed by the ten-eleven translocation (TET) [46,47]. 5-hmC can act as an intermediate in DNA demethylation or as an independent epigenetic marker [48,49]. Recent findings suggest that DNMTs contribute to 5-hmC production, implicating this modification in cellular development, pluripotency, ageing, and cancer [44,50,51].

### 2.3. Histone Modifications

#### 2.3.1. Histone Acetylation

Among histone modifications, acetylation is a well-studied phenomenon in which N-terminal lysines of histones undergo reversible acetylation and deacetylation in response to various physiological conditions [52,53]. Histone acetylation is generally associated with increased gene transcription and is typically observed in regions containing actively transcribed genes [54,55]. This post-translational modification results in a more relaxed chromatin structure, facilitating transcriptional activation at specific genomic loci [56]. The regulation of histone acetylation involves two classes of enzymes: histone acetyltransferases (HATs) and histone deacetylases (HDACs). HATs catalyze the addition of acetyl groups to lysine residues on histone tails, which neutralizes their positive charge. This neutralization reduces the affinity between histones and DNA, resulting in a less condensed chromatin structure, particularly in the promoter regions of genes, making them more accessible to enhancers and RNA polymerases [57,58]. Conversely, HDACs are associated with transcriptionally inactive regions of the genome. By removing acetyl groups from lysine residues, HDACs increase the positive charge on histone tails, thereby strengthening the histone–DNA interaction. This modification leads to a more compact chromatin state, thus limiting the accessibility of transcriptional regulators to the DNA template [54,59].

#### 2.3.2. Histone Methylation

Histone methylation involves the addition of one to three methyl groups to specific amino acids—particularly lysine and arginine residues—in the tails of histone proteins H3 and H4 [60,61,62]. This modification can influence the recruitment and binding of transcription factors to targeted genes. Histone methylation is catalyzed by lysine and arginine methyltransferases, while demethylation is facilitated by histone demethylases [63]. Polycomb group proteins (PcG) are a family of enzymes that regulate histone methylation through their roles in Polycomb repressive complexes (PRCs), leading to more compact chromatin and gene silencing [64]. It has been observed that PcG proteins collaborate with DNMTs to enhance gene repression and maintain methylation patterns [30,65]. The biological outcomes of histone methylation depend highly on the modified residues [66,67]. For instance, the methylation of lysine residues 4, 36, and 79 on histone H3 (H3K4, H3K36, and H3K79) and lysine 20 on histone H4 (H4K20) are generally associated with transcriptional activation. Conversely, the trimethylation of lysine residues 9 and 27 on histone H3 (H3K9me3 and H3K27me3) is associated with gene silencing [67].

#### 2.3.3. Histone Phosphorylation

Histone phosphorylation is regulated by kinases and phosphatases that add and remove phosphate groups predominantly at serine, threonine, and tyrosine residues [68]. This modification alters the electric charge of histones, thereby modulating chromatin structure and regulating gene expression [69]. Additionally, histone phosphorylation interacts with other histone modifications, facilitating cross-talk between regulatory mechanisms, including DNA repair and damage responses [70].

#### 2.3.4. Histone Ubiquitination

Ubiquitination is a post-translational modification process in which a small regulatory protein called ubiquitin is covalently attached to the target histone protein, predominantly at lysine residues [71,72]. This modification is primarily associated with protein degradation via the ubiquitin–proteasome system (UPS), crucial in maintaining protein stability and function [73]. The ubiquitination process involves a sequential enzymatic cascade, including ubiquitin-activating enzymes (E1), ubiquitin-conjugating enzymes (E2), and ubiquitin-protein ligases (E3). Conversely, the removal of ubiquitin is catalyzed by deubiquitinating enzymes, a subclass of isopeptidases that hydrolyze the peptide bond between ubiquitin and its histone substrate [74]. Histones can undergo either mono- or poly-ubiquitination, each impacting chromatin activity differently [75]. Generally, the monoubiquitination of histone H2A is involved in gene silencing, while the monoubiquitination of histone H2B is typically linked to transcriptional activation [32].

#### 2.3.5. Histone Sumoylation

Sumoylation is an essential post-translational modification involved in various cellular processes, including transcription, DNA replication, cell cycle progression, and stress response [76]. Histones are substrates for sumoylation, which regulates chromatin dynamics and gene expression. Small ubiquitin-like modifier (SUMO) proteins are conserved across all eukaryotes and modify target proteins’ functions through sumoylation [77]. Humans express five SUMO paralogs: SUMO-1, -2, -3, -4, and -5, which are produced as inactive precursors and processed into their active forms. Active SUMO proteins attach to lysine residues on target proteins through a series of enzymatic steps similar to ubiquitination [78]. Key molecules involved in sumoylation include ubiquitin-conjugating enzyme 9 (UBC9) and the E3 SUMO ligase PIASy, which can bind to promoter regions [79]. Recent findings suggest that histone sumoylation can both disrupt higher-order chromatin structures and recruit factors involved in gene activation or repression [80].

### 2.4. Non-Coding RNAs (ncRNAs)

Mature miRNAs are significant components of the epigenetic landscape. The human genome potentially encodes over 1000 miRNAs that regulate the expression of approximately 30% of all protein-coding genes [81]. A single miRNA can influence the production of hundreds of different proteins, while multiple miRNAs can regulate the expression of a particular protein [32]. MiRNAs bind to complementary sequences in the 3′ untranslated region 3′ UTR of messenger RNAs (mRNAs), leading to gene silencing via mRNA cleavage, destabilization through the deadenylation of the poly(A) tail, and the inhibition of mRNA translation [81,82]. Furthermore, miRNAs can target key enzymes responsible for epigenetic modifications, such as DNMTs, HDACs, and histone methyltransferases [81,82]. Moreover, the expression of miRNAs is regulated by epigenetic mechanisms, including DNA methylation and histone modifications [81,82]. This reciprocal relationship between miRNAs and epigenetic regulation forms the miRNA–epigenetic feedback loop, which has emerged as a novel mechanism for regulating cellular processes, such as cell proliferation, apoptosis, and differentiation [81,82].

In addition to miRNAs, other ncRNAs influence gene transcription, too. Long non-coding RNAs (lncRNAs) regulate gene expression at various levels, including the transcriptional, post-transcriptional, and post-translational stages [83,84,85]. Researchers suggest that lncRNAs play a targeting role by binding to specific methyltransferases and demethylases, directing these enzymes to particular genomic locations to control gene expression [86]. Furthermore, lncRNAs can form circular RNAs (circRNAs), which function similarly to miRNAs by impacting DNA methylation, the immune response, and RNA processing [87]. CircRNAs can also bind to specific miRNAs, serving as competitive inhibitors to prevent these miRNAs from interacting with their target mRNAs [88]. All the aforementioned epigenetic mechanisms are summarized in Table 1.

## 3. Epigenetic Regulation in Skin Homeostasis

Epidermal homeostasis refers to the delicate equilibrium among epidermal cells’ proliferation, differentiation, and apoptosis, essential for maintaining tissue organization and proper function [89,90]. In most regions of the adult human body, the epidermis undergoes renewal approximately every month. This renewal process relies on a well-orchestrated balance between producing new epidermal cells in the basal layer and exfoliating cells from the stratum corneum [91]. Various factors, including the developmental stage and potential external injuries, influence the rate at which adult epidermal SCs renew themselves [92]. Epigenomics is a critical regulator in skin homeostasis, a field encompassing mechanisms that can alter gene expression profiles without changing the DNA sequence [93]. Specifically, epigenetic regulation and preserving epidermal homeostasis involve mechanisms that modify DNA methylation and participate in chromatin remodelling [94,95]. Additionally, miRNAs play a significant role, as they are estimated to regulate the post-transcriptional control of up to 60% of all expressed genes [96].

### 3.1. The Role of DNA Methylation in Skin Homeostasis 

DNA methylation is the first epigenetic mechanism that plays a pivotal role in skin maintenance. During this process, a -CH_3_ is transferred, typically from S-adenosylmethionine (SAM), to a cytosine located at the 5′ position of the pyrimidine ring in the DNA molecule [94]. This methylation predominantly occurs at CpG sites, known as CpG islands, and the transfer is mediated by DNMTs, including DNMT1, DNMT3A, and DNMT3B [41,97]. Specifically, it has been demonstrated through the cleavage of specific regulatory domains in mammalian DNMTs followed by protein sequencing in murine erythroleukemia cells that DNMT1 replicates methylation patterns on newly synthesized DNA by preferentially recognizing hemimethylated CpG nucleotides over unmethylated sequences [98]. Thus, DNMT1 is the primary maintenance methyltransferase in vivo, preserving DNA methylation patterns after each cell division. The importance of DNMT1 is underscored by experiments involving gene targeting in mouse embryonic SCs, where its silencing resulted in the widespread demethylation of all analyzed sequences [99]. In contrast, DNMT3A and DNMT3B are de novo methyltransferases, establishing new methylation patterns [100]. Their role in de novo methylation is highlighted by gene targeting studies in embryonic SCs, where the inactivation of DNMT3A and DNMT3B disrupts normal de novo methylation processes [100].

Among these methyltransferases, DNMT1 is typically upregulated in the epidermal progenitor SCs residing in the basal layer [101]. Interestingly, its complete absence is evident in the outer layers characterized by more differentiated keratinocytes. This indicates that DNMT1’s contribution is crucial for maintaining the proliferative capacity of basal SCs and, consequently, for suppressing their terminal differentiation. The above data were acquired from microarray analysis, a procedure used to identify genes modified during calcium-induced differentiation. In parallel, immunohistochemistry results demonstrated that DNMT1 deficiency leads to the suppression of proliferation-related genes and the upregulation of genes necessary for differentiation, revealing these SCs’ inability to maintain their self-renewal capacity and progression towards differentiation. Notably, several genes and transcription factors, including S100 calcium-binding protein P (S100P), late cornified envelope 3D (LCE3D), MAF bZIP transcription factor F (MAFF), specificity protein 1 (SP1), and POU class 2 homeobox 3 (POU2F3), were found to lose their methyl groups as differentiation progressed [102,103,104,105]. Changes in methylation patterns were detected using techniques such as methylated DNA immunoprecipitation (MeDIP) and MeDIP-coupled quantitative PCR (QPCR) [101].

Associated with the activity of DNMT1 at hemimethylated CpG dinucleotides is the ubiquitin-like with PHD and ring finger domains 1 (UHRF1) protein [106]. This protein facilitates the interaction of DNMT1 with hemimethylated DNA, resulting in a common localization and similar effects on cell differentiation [107]. In contrast to DNMT1 and UHRF1, growth arrest and DNA-damage-inducible protein 45 alpha (Gadd45a) promotes differentiated cell fate over progenitor expansion by participating in the demethylation process [101,108,109]. Besides preserving the self-renewal capacity of basal SCs, DNMT1 has also been implicated in the induction of epidermal autoinflammation [110]. Specifically, a histological examination of DNMT1 knockout mice revealed significant structural changes, including dermal thinning and extensive immune cell infiltration. These modifications were attributed to disruptions in the cGAS/STING pathway, which is responsible for the expression of interferon-stimulated genes (ISGs) in response to foreign DNA or chromosomal instability. Additionally, the activation of toll-like receptors (TLRs) and the inflammasome contributed to these alterations in tissue organization [111,112].

**Figure 1:**
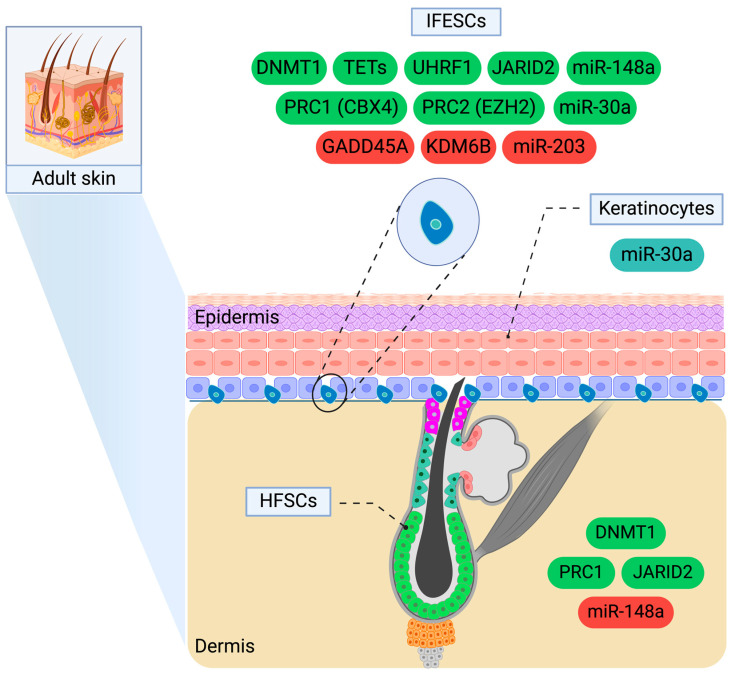
The epigenetic regulators of skin homeostasis. DNMT1, TETs, UHRF1, JARID2, PRC1 (CBX4), PRC2 (EZH2), GADD45A, KDM6B, miR-30a, miR-148a, and miR-203 control the function of stem cells residing in the basal layer. Specifically, green-coloured factors enhance the proliferative capacity of IFESCs while inhibiting their differentiation. Conversely, red-coloured molecules reduce the proliferative dynamics of IFESCs and promote their differentiation. DNMT1, PRC1, and JARID2 promote the expansion of HFSCs, whereas miR-148a exerts an opposing effect. The apoptosis of keratinocytes is mediated by miR-30a. IFESCs: interfollicular stem cells, HFSCs: hair follicle stem cells, CBX4: chromobox 4, DNMT1: DNA methyltransferase 1, EZH2: enhancer of zeste homolog 2, GADD45A: DNA-damage-inducible protein 45 alpha, JARID2: Jumonji and AT-rich interaction domain containing 2, KDM6B: lysine demethylase 6B, miR-30a: microRNA-30a, miR-148a: microRNA-148a, miR-203: microRNA-203, PRC1: Polycomb repressive complex 1, PRC2: Polycomb repressive complex 2, TETs: ten-eleven translocation family proteins, UHRF1: ubiquitin-like with PHD and ring finger domains 1. Created with BioRender.com.

DNMT1 plays a critical role in the homeostasis of adult HFs (Figure 1) [113]. It is expressed in the HF matrix, outer and inner root sheaths during anagen, as well as in the hair germ during telogen. Mice with mutations in the gene encoding DNMT1 exhibited progressive alopecia, characterized by a gradual reduction in hair fibres and HFs, alongside significant variability in HF size and an expanded hair canal. Hair bulge SCs remained relatively stable and could be activated to develop into hair germs and produce hairs. However, the activation process was less efficient, and their capacity for self-renewal was impaired. Regarding DNMT3A and DNMT3B, they appear to play a protective role in the epidermis against tumour formation [114]. Their absence in the mouse epidermis leads to squamous transformation. Histological analyses from knockout mice indicated that the loss of Dnmt3a triggers tumorigenesis through the upregulation of the adipogenesis regulator PPARγ, while the absence of Dnmt3b facilitates tumour growth and metastasis. However, both methyltransferases seem inessential for maintaining epidermal homeostasis, as knockout mice did not display significant alterations in their epidermis compared to wild-type cohorts.

In contrast to DNMTs, which mediate methylation, TET enzymes are responsible for removing methyl groups via oxidation, thus making the methylation process reversible [115]. Specifically, the TET family includes TET1, TET2, and TET3 enzymes, which catalyze the oxidation of 5-methylcytosine to 5-hydroxymethylcytosine, 5-formylcytosine, and 5-carboxycytosine [116,117,118,119]. A delicate balance in the expression of genes encoding DNMTs and TET enzymes is essential for preserving proper methylation patterns. The diminished expression of TET1 and TET2 is associated with skin diseases such as psoriasis. Reduced TET expression may impair the self-renewal capacity of basal SCs (Figure 1), leading to the expansion of the transit-amplifying cells’ (TACs’) compartment [120]. TACs, descendants of SCs, are destined to exit the cell cycle and undergo terminal differentiation. Observations in both human and murine models of psoriasis suggest that reduced methylation levels may influence the expression of genes critical for regulating basal SC homeostasis and responses to proliferative signals. Immunohistochemical analyses indicated that more SCs undergo asymmetric division in mouse models and human epidermis affected by psoriasis [121]. This hypomethylation, resulting from aberrant TET expression, appears to trigger the expansion of the TACs’ compartment, which is characteristic of psoriasis [120,122].

### 3.2. Histone Modification Dynamics and Their Impact in Skin Maintenance

Post-translational modifications of histone residues represent another critical epigenetic mechanism involved in skin homeostasis [123]. Key regulators of this epigenetic mechanism are the PcG proteins, which act as transcriptional repressors and play a crucial role in regulating cell lineage decisions during homeostasis [124]. These proteins form complexes known as PRC1 and PRC2, responsible for chromatin condensation and, thus, transcriptional repression [125,126]. Specifically, chromobox 4 (Cbx4), a subunit of the PRC1 complex, is essential for maintaining the multipotency of basal SCs and preventing their terminal differentiation [127]. This is evidenced by studies in which the deletion of Cbx4 in mouse epidermis resulted in decreased basal SC proliferation and increased premature differentiation. Similarly, the absence of Cbx4 in human basal SCs leads to the acquisition of terminally differentiated morphology and increased susceptibility to cellular ageing.

HF homeostasis is also influenced by the PRC1 [128]. Recent studies have demonstrated that the loss of PRC1 activity in Lgr5^+^ hair follicle stem cells (HFSCs) results in decreased levels of H2AK119Ub and a significant delay in HF regeneration, potentially due to the reduced proliferation rates of HFSCs. Concerning the PRC2, it comprises the embryonic ectoderm development (EED) protein, suppressor of zeste 12 (SUZ12) and enhancer of zeste homolog 1/2 (EZH1/2) [129]. The PRC2 catalyzes the H3K27me3. This epigenetic modification is crucial for determining the balance between basal SCs with proliferative capacity and differentiating keratinocytes [130]. Specifically, Ezh2 expression inversely correlates with the differentiation status of keratinocytes in mice, with its levels being higher in basal SCs and gradually decreasing as differentiation proceeds [131]. Consequently, Ezh2 appears to play a role in repressing the expression of genes associated with differentiation in the basal epidermis [65].

Jumonji and AT-rich interaction domain containing 2 (Jarid2) is another auxiliary molecule responsible for the recruitment of the PRC2 complex and is crucial for the homeostasis of the mouse epidermis [132]. It is essential for postnatally maintaining the proper balance between epidermal SCs and the activation of HFSCs [133]. Additionally, the loss of Jarid2 leads to delayed HF cycling due to reduced proliferation of HFSCs and their progeny. Immunohistochemistry in mouse keratinocytes has shown that, although the deletion of Jarid2 in the epidermis does not impact its development during the embryonic stage, it decreases the proliferative capacity of basal SCs while promoting their differentiation postnatally. The methylation mediated by PRC2 is reversible, as specific demethylases can remove methyl groups [134]. Lysine demethylase 6B (KDM6B), a demethylase that counteracts the enzymatic activity of PRC2, enhances gene expression in SC populations and regulates processes, including the cell cycle, differentiation, and apoptosis [135]. KDM6B’s enzymatic activity depends on the JmjC domain at the C-terminal of the protein, which serves as the catalytic centre responsible for removing repressive H3K27me3 epigenetic marks from promoters and gene bodies. Specifically, KDM6B activation triggers premature epidermal differentiation, while the depletion of KDM6B prevents the activation of differentiation-related genes in human keratinocytes [10,134].

### 3.3. The Influence of miRNAs on Skin Homeostasis

An alternative mechanism of the epigenetic post-transcriptional regulation of epidermal homeostasis involves miRNAs. These RNA-targeting molecules are non-coding RNAs consisting of approximately twenty-two nucleotides and play a crucial role in repressing gene expression [136]. MiR-203 is a key regulator of skin homeostasis, as it promotes the differentiation of basal SCs [137]. In mouse models, the overexpression of miR-203 results in basal SCs’ early differentiation and reduces their proliferative capacity [138]. Conversely, the ablation or reduced levels of miR-203 are associated with increased proliferative capacity, extending beyond the basal layer of the epidermis [137]. Interestingly, the gene expression outcomes arise from an intricate interaction between miR-203 and one of its mRNA targets, p63 [139]. p63 is highly concentrated in the basal layer of the epidermis and serves as a specific marker for SCs, playing a role in controlling both their proliferation and differentiation. The immunohistochemical detection of p63 in human epidermal keratinocytes indicates a high proliferative rate of SCs, whereas reduced p63 expression correlates with the initiation of terminal differentiation in the adult epidermis [140,141]. Given that p63 is a direct target of miR-203, its expression is upregulated in the absence of miR-203 and suppressed in the presence of miR-203, thus contributing to the differentiation process [138].

An additional miRNA implicated in human skin homeostasis is miR-30a. Research conducted in human keratinocytes confirms that, in addition to its role in cell ageing, its upregulation hinders epidermal differentiation through the repression of lysyl oxidase (LOX), a gene involved in keratinocyte differentiation [142,143,144]. Moreover, miR-30a overexpression disrupts epidermal barrier function, as confirmed by the Lucifer yellow assay, which evaluates outside-in permeability, and the trans-epidermal water loss (TEWL) test, which assesses inside-out permeability [144]. Another effect of miR-30a upregulation is the enhancement of apoptosis in keratinocytes by inactivating apoptosis and caspase activation inhibitor (*AVEN*), a gene encoding an apoptotic inhibitor. MiR-148a is another epigenetic regulator crucial for maintaining epidermis and HFs [145]. It is involved in preserving skin integrity, with its absence correlated with the premature differentiation of keratinocytes. MiR-148a orchestrates the balance of cellular processes necessary for developing the mouse epidermis and HFs by modulating the activity of two gene transcripts, E74-like ETS transcription factor 5 (Elf5) and Rho-associated coiled-coil containing protein kinase 1 (Rock1). Notably, in HFs, miR-148a is expressed spatiotemporally, primarily during the telogen phase, when the HF is inactive, and the hair has completed its growth. The inhibition of miR-148a drives HFs into the anagen stage, characterized by the production of new hair.

## 4. Epigenetic Landscape of Skin Ageing

### 4.1. Insights into the Ageing Process

Ageing is an inevitable and intricate biological process that influences the entire organism, affecting every cell type and impairing its functions. At the cellular level, it leads to a gradual deterioration of all organelles, diminishing the cell’s capacity for self-regulation, maintaining homeostasis, and replication. This decline extends to all organ systems, resulting in a progressive reduction in physiological function throughout the body [146,147]. Closely associated with ageing is the activation of the senescence programme, which involves the programmed degeneration of cellular components and cell cycle arrest. Specifically, cellular senescence refers to a state that cells enter in response to stressful conditions or certain physiological events. It is marked by a prolonged, often irreversible halt in the cell cycle, along with changes in secretion, macromolecular damage, and metabolism [148]. Four key characteristics define senescent cells, namely (1) withdrawal from the cell cycle, (2) damage to macromolecules, (3) a secretory phenotype known as the senescence-associated secretory phenotype (SASP), and (4) disrupted metabolic activity. These cells release a wide range of substances, including pro-inflammatory cytokines, chemokines, growth factors, angiogenic factors, and matrix metalloproteinases, which are collectively referred to as SASP. In this context, epigenetics has been found to play a critical role in the regulation and programming of senescence [149].

The skin, serving as the boundary between the human body and the external environment, is not exempt from ageing. Various factors contribute to this process, including increased levels of reactive oxygen species, UV radiation, smoke, environmental chemicals, diet, and hormonal changes [150,151,152,153]. Establishing cellular senescence plays a central role in the gradual deterioration of skin health, as many senescent cells accumulate in aged skin. This decline is characterized by several biological landmarks, the most prominent being genomic instability, telomere shortening, and alterations in epigenetic expression patterns [154,155]. Beyond these changes in genetic material maintenance, ageing cells exhibit impaired nutrient-sensing mechanisms, mitochondrial and protein dysfunction, disrupted macroautophagy, and diminished capacity for cellular self-renewal. Additionally, inflammation and dysbiosis are commonly observed [156,157]. Phenotypically, skin ageing is manifested by thinning skin, wrinkle formation, and the deterioration of HFs, which is evidenced by brittle and greying hair [158,159,160,161,162].

### 4.2. Epigenetic Mechanisms Regulating Ageing in Fibroblasts

Epigenetic regulation plays a significant role in the age-related deterioration of skin cells [163,164]. Notably, the accumulation of senescent dermal fibroblasts (FBs)—the primary cells in the dermis responsible for producing key connective tissue components, such as collagen and elastic fibres—is strongly implicated in skin ageing [165,166]. Age-related changes in the dermis mainly arise from the dysfunction of FBs that accumulate damage over time and lose their ability to remodel and organize the extracellular matrix (ECM). This leads to reduced synthesis of collagen and elastin [167]. Collagen atrophy and the loss of elastic fibres are critical components of skin ageing, underscoring the essential role of FBs in maintaining skin elasticity [168]. Using micrococcal nuclease digestion and electron microscopy techniques to monitor age-related changes in these cells, researchers observed that human skin FBs derived from older donors, in contrast to younger ones, exhibited alterations in their chromatin structure. Specifically, the spacing between nucleosomes was found to be irregular [169]. These findings indicate that with ageing, the length of linker DNA between nucleosomes becomes increasingly variable, potentially contributing to FB dysfunction and impaired ECM deposition [165,169,170].

As the senescence programme is activated in FBs, its progression is further driven by the accumulation of heterochromatin structures known as senescence-associated heterochromatin foci (SAHF), resulting from the activation of the p16INK4a/pRb pathway [171]. The loci mentioned above, co-localized with the epigenetic marker H3K9me3, along with HP1 and the retinoblastoma (RB) protein, repress E2F target genes essential for cell proliferation, thereby establishing a stable senescent state [171,172]. Various enzymes play a pivotal role in epigenetic regulatory networks. DNMT1, responsible for the methylation of CpG islands, is significantly involved in the epigenetic regulation of dermal FBs and the maintenance of skin cells, primarily through the repression of the INK4a/ARF locus [101,173]. Levels of DNMT1 are reduced in ageing FBs, which leads to the induction of cellular senescence in human skin [174,175]. The decreased levels of DNMT1 likely contribute to the reduced deposition of new collagen in the ECM and the degradation of pre-existing collagen, thereby impacting skin ageing [176,177].

**Figure 2:**
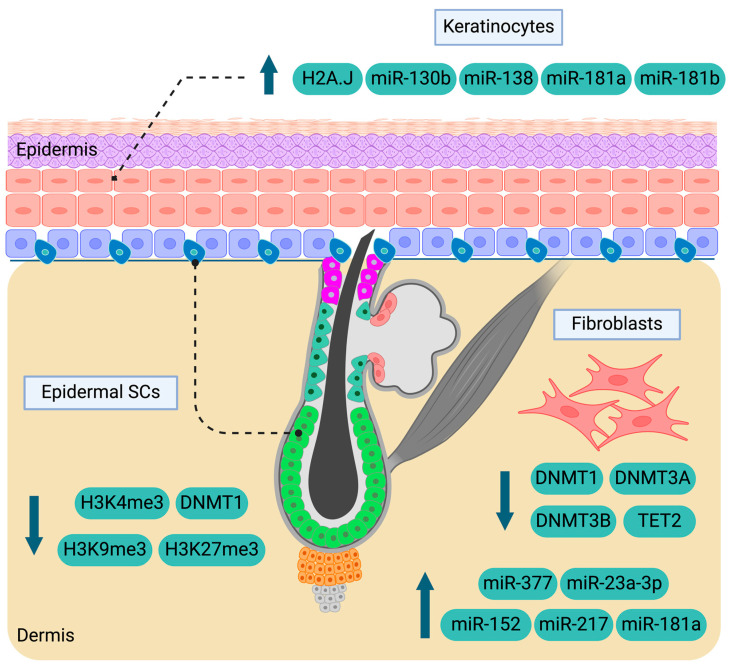
Epigenetic mediators and marks implicated in aged skin. The effectors of fibroblasts are DNMT1, DNMT3A, DNMT3B, TET2, and several miRNAs. H2A.J, miR-130b, miR-138, miR-181a and miR-181b regulate the biological behaviour of keratinocytes, while H3K9me3, H3K4me3, H3K27me3 and DNMT1 act in epidermal SCs. Upward-pointing arrows indicate elevated levels of the corresponding molecules or marks in aged skin, whereas downward-pointing arrows represent reduced levels. SCs: stem cells, miRNAs: microRNAs, DNMT1: DNA methyltransferase 1, DNMT3A: DNA methyltransferase 3A, DNMT3B: DNA methyltransferase 3B, H2A.J: Histone H2A variant J, H3K27me3: trimethylation of lysine 27 on histone H3, H3K4me3: trimethylation of lysine 4 on histone H3, H3K9me3: trimethylation of lysine 9 on histone H3, TET2: ten-eleven translocation methylcytosine dioxygenase 2. Created with BioRender.com.

Regarding the regulation of DNMT1, it has been demonstrated that DNMT1 expression in human skin FBs is downregulated by miR-377, a process that induces senescence in these cells (Figure 2). This is supported by the observation that miR-377 levels are significantly elevated in passage-aged human skin FBs [178]. Similarly, miR-217 has been shown to suppress DNMT1 expression in human skin FBs directly, and the overexpression of miR-217 induces a senescence-like state in FBs derived from young patients [173]. Furthermore, it is crucial to note that the concentrations of other enzymes involved in the methylation and demethylation of DNA are altered with skin ageing [179]. Western blot analysis in mice revealed that with skin ageing, the expression levels of DNMT3a, DNMT3b, and Tet2 decrease significantly, with DNMT3b exhibiting a more drastic decline compared to DNMT3a, while DNMT1 and Tet1 levels remain relatively stable [180]. Additionally, alterations in DNA methylation patterns in gene promoters occur as the skin ages. A whole-genome-promoter methylation array study documented increased and decreased DNA methylation in gene promoters with ageing. Hypermethylated genes were primarily associated with cell proliferation and growth, whereas hypomethylated genes were enriched in categories related to responses to stimuli, such as genes encoding receptor proteins [180].

DNMT3A has been further implicated in the ageing process of dermal FBs through its regulation of the *LOXL1* gene, a gene whose protein product is involved in the maturation of elastic fibres (Figure 2) [180,181,182,183,184]. Researchers reached this conclusion by performing anti-DNMT3A chromatin immunoprecipitation in human skin FBs from younger and older individuals [181]. It was identified that in FBs from older subjects, DNMT3A protein exhibited greater binding to the *LOXL1* promoter, accompanied by the increased overall methylation of the *LOXL1* promoter. The hypermethylation was correlated with decreased LOXL1 mRNA levels, indicating the reduced transcriptional activity of *LOXL1* in aged skin [181]. While it is widely accepted that ageing is associated with a general reduction in DNA methylation, there is also a simultaneous occurrence of the localized hypermethylation of CpG islands in the promoters of specific genes, particularly tumour suppressor genes, leading to their transcriptional silencing. Notable among these genes are *LOX, p16INK4a*, *RUNX3,* and *TIG1* [185,186,187,188]. This evidence supports a strong connection between DNMT3A levels, the methylation state of ageing-associated genes, and cell cycle regulation. It also underscores the impact of these epigenetic changes on the connective tissue and skin repair processes that deteriorate with ageing.

In addition to their correlation with DNMT1 levels, the regulatory significance of miRNAs is further demonstrated by their role as cargo in extracellular vesicles (EVs) secreted by dermal FBs [189]. EVs are cell-derived membranous particles that mediate intercellular communication among various types of skin cells, including FBs, keratinocytes, melanocytes, and HF cells. These vesicles contain a heterogeneous array of molecules, including miRNAs, proteins, and lipids [190,191,192,193,194]. When FBs enter a senescent state, EVs and their miRNA content act as key mediators in establishing the SASP in human skin FBs. SASP, a state acquired by senescent cells, enables them to secrete extracellular modulators that reinforce senescence-induced cell cycle arrest, contribute to developing a pro-inflammatory environment in skin tissue, and potentially facilitate skin ageing [189,195,196]. One particularly significant miRNA derived from FBs is miR-23a-3p [189]. This miRNA targets hyaluronan synthase 2 (HAS2) and plays a substantial role as an effector in the induction of senescence and dermal ageing [197]. The expression of miR-23a-3p is upregulated in aged and senescent FBs, leading to the inhibition of HAS2. As a result, hyaluronic acid levels in the ECM are reduced, leading to decreased skin hydration and elasticity [197,198].

In the same context, the increased expression of miR-152 and miR-181a in senescent human skin FBs has been observed, and their overexpression is sufficient to induce cellular senescence [199,200,201]. The consequent reduction in the expression of integrin α5 and collagen XVI, along with alterations in the ECM structure, is consistent with the compositional changes in the skin during ageing [200]. Several other miRNAs have been identified as potential biomarkers of ageing in human dermal senescent FBs. These include 15 miRNAs with upregulated levels, such as let-7d-5p, let-7e-5p, miR-23a-3p, miR-34a-5p, miR-122-5p, miR-125a-3p, miR-125a-5p, miR-125b-5p, miR-181a-5p, miR-221-3p, miR-222-3p, miR-503-5p, miR-574-3p, miR-574-5p, and miR-4454 [202]. Consequently, these miRNAs exhibit a strong correlation with the ageing process, although their precise biological roles remain to be elucidated [202]. Furthermore, an RNA microarray performed on UVA-irradiated, photoaged skin cells revealed the upregulation of miR-365, miR-30b, miR-148a, miR-30c, and miR-199a-5p and the downregulation of miR-1246, miR-146a, miR-3613-3p, miR-218, miR-146b-5p, miR-4281, and miR-181 compared to untreated cells [203]. This suggests a potential role for these miRNAs in photo-induced ageing.

### 4.3. Ageing Dynamics and Keratinocytes

Keratinocytes, the predominant cells of the epidermis, also exhibit age-related epigenetic changes. Although UV radiation is the most detrimental factor contributing to skin damage and accelerating ageing, keratinocytes in photo-protected areas also display age-associated epigenetic alterations [204,205,206,207]. Recent studies on photo-protected skin from healthy individuals have demonstrated a significant accumulation of H2A.J-positive keratinocytes as ageing progresses (Figure 2) [206]. H2A.J, a histone variant, is found in elevated levels in keratinocytes derived from older individuals compared to those derived from younger ones [206]. Regarding microRNAs expressed in human skin keratinocytes, miR-130b, miR-138, miR-181a, and miR-181b are associated with the induction of replicative senescence [199]. These microRNAs modulate the levels of regulatory proteins such as p63 and SIRT1, which play critical roles in skin homeostasis, cell proliferation, and the manifestation of ageing phenotypes. Furthermore, the expression of these senescence-inducing microRNAs is directly inhibited by p63 in a negative feedback loop [199]. Consequently, miR-130b, miR-138, miR-181a, and miR-181b strongly correlate with the initiation of senescence and possibly the ageing process [202,208]. SIRT1, a NAD-dependent histone deacetylase, is associated with extended longevity in mammals and delayed ageing when overexpressed. This correlation is further supported by experiments involving transgenic mice overexpressing SIRT1, which demonstrate delayed ageing phenotypes and increased lifespan [209,210].

### 4.4. Epigenetic Modulation of Ageing in Epidermal SCs

Epidermal SCs undergo significant epigenetic modifications during ageing, profoundly affecting their renewal capabilities (Figure 2). Among these epigenetic changes, histone post-translational modifications are critical in regulating ageing. Specifically, disruptions in histone methylation and acetylation patterns can significantly impact the maintenance of SC properties [211,212]. A recent study on murine HFSCs revealed a marked decrease in chromatin accessibility in aged HFSCs, particularly in genes essential for self-renewal and differentiation [213]. Furthermore, aged HFSCs exhibited a reduced capacity to activate bivalent genes—meaning genes whose promoters/enhancers have both repressing and activating epigenetic regulators in the same region—which are crucial for maintaining their renewal potential [213]. Notably, the H3K9me3 and trimethylation of histone H3 at lysine 4 (H3K4me3) are essential modifications that regulate the balance between quiescence and proliferation in HFSCs [214].

Aside from histone modifications, DNA methylation and demethylation enzymes are critical for maintaining epidermal SCs. As we have already mentioned, DNMT1 is essential for sustaining the self-renewal potential of progenitor cells in the human epidermis and preventing their differentiation. However, the precise biological mechanism remains unclear [101,107]. The importance of DNMT1 is further highlighted by experiments involving regenerated human skin on immunodeficient mice, where low levels of DNMT1 correlate with hypoplastic tissue and increased tissue loss [101]. Additionally, in murine models, the targeted deletion of DNMT1 in the epidermis leads to age-associated changes such as HF miniaturization, an extended telogen phase, hair loss, and hyperplasia of both the epidermis and sebaceous glands [113]. This evidence underscores the pivotal role of DNA methylation in regulating and preserving epidermal and HFSC function.

## 5. Conclusions and Prospects

Epigenetic regulation assumes a central role in skin homeostasis and ageing. Key epigenetic modifications, such as DNA methylation, histone alterations, and non-coding RNAs, profoundly influence gene expression patterns that govern the proliferation and differentiation of skin cells. The modulation of these epigenetic marks impacts critical pathways essential for preserving skin integrity and function over time. Despite significant advancements in mapping these modifications, the intricate mechanisms and their functional consequences remain incompletely understood. Given the skin’s heterogeneous cellular composition, elucidating the specific epigenetic changes within each cell type and their distinct contributions to skin maintenance and ageing requires further investigation. Single-cell epigenetic studies offer a promising approach to address this complexity. Furthermore, the interplay between genetic predispositions, environmental influences, lifestyle choices, and epigenetic modifications forms an intricate network that still needs to be fully elucidated. Particularly noteworthy is the emerging role of non-coding RNAs, especially lncRNAs, whose functions are largely unexplored. Bridging foundational epigenetic research with clinical applications remains a formidable challenge. Identifying reliable epigenetic biomarkers for skin ageing and developing therapeutic interventions to modulate these marks hold significant promise. Addressing these research gaps through interdisciplinary collaboration and advanced technologies will pave the way for innovative therapeutic strategies and a deeper understanding of the role of epigenetics in skin homeostasis and ageing.

## Figures and Tables

**Table 1:** Overview of epigenetic mechanisms and their biological impact.

Epigenetic Mechanism	Key Modifiers	Effect of Modification
DNA methylation	DNA methyltransferases	Transcriptional repression; Gene silencing
DNA hydroxymethylation	Ten-eleven translocation enzymes	Regulating gene expression; Suppressing or activating genes
Histone acetylation	Histone acetyltransferases; Histone deacetylases	Histone charge regulation; Transcriptional activation or repression
Histone methylation	Lysine and arginine methyltransferases; Histone demethylases	Transcriptional activation or gene silencing, depending on the specific residue being modified
Histone phosphorylation	Kinases and phosphatases	Modulation of chromatin structure; Gene silencing or activation
Histone ubiquitination	Ubiquitin-activating enzymes; Ubiquitin-conjugating enzymes; Ubiquitin-protein ligases; Deubiquitinating enzymes	Gene silencing or activation; Mono- or poly-ubiquitination of specific lysine residues
Histone sumoylation	Ubiquitin-conjugating enzyme 9; E3 SUMO ligase	Ubiquitination-like enzymatic pathway; Positive and negative effects on gene expression
Non-coding RNAs	miRNA	mRNA cleavage; Deadenylation of the poly(A) tail; Inhibition of mRNA translation; miRNA-epigenetic feedback loop;
	lncRNAs	Binding to specific methyltransferases and demethylases; Form circular RNAs
	circRNAs	Impacting DNA methylation and RNA processing; Binding to specific miRNAs

circRNAs: circular RNAs; DNA: deoxyribonucleic acid; E3: third enzyme in ubiquitin or SUMO ligase complex; lncRNAs: long non-coding RNAs; miRNA: microRNA; mRNA: messenger RNA; RNA: ribonucleic acid; SUMO: small ubiquitin-like modifier.

## Data Availability

Not applicable.

## Abbreviations

AVEN	Apoptosis and caspase activation inhibitor
CBX4	Chromobox 4
CH_3_	Methyl group
circRNA	CircularRNA
CpG	Cytosine–phosphate–guanine
DNA	Deoxyribonucleic acid
DNMT	DNA methyltransferase
ECM	Extracellular matrix
EED	Embryonic ectoderm development
ELF5	E74-like ETS transcription factor 5
EMD	Emerin
EV	Extracellular vesicle
EZH1/2	Enhancer of zeste homolog 1/2
FB	Fibroblast
GADD45A	DNA-damage-inducible protein 45 alpha
H2A	Histone 2A
H2AK119Ub	Histone 2A lysine 119 ubiquitination
H2B	Histone 2B
H3	Histone H3
H3K27me3	Histone 3 lysine 27 trimethylation
H3K36	Histone 3 lysine 36
H3K4	Histone 3 lysine 4
H3K4me3	Trimethylation of histone H3 lysine 4
H3K79	Histone 3 lysine 79
H3K9me3	Histone 3 lysine 9 trimethylation
H4	Histone H4
H4K20	Histone 4 lysine 20
HAS2	Hyaluronan synthase 2
HATs	Histone acetyltransferases
HDACs	Histone deacetylases
HFs	Hair follicles
HFSCs	Hair follicle stem cells
HP1	Heterochromatin protein 1
ISGs	Interferon-stimulated genes
JARID2	Jumonji and AT-rich interaction domain containing 2
KDM6B	Lysine demethylase 6B
LCE3D	Late cornified envelope 3D
lncRNA	Long non-coding RNA
LOX	Lysyl oxidase
MAFF	MAF bZIP transcription factor F
MBDs	Methyl-CpG-binding domain proteins
meDIP	Methylated DNA immunoprecipitation
miRNAs	MicroRNAs
mRNA	Messenger RNA
ncRNA	Non-coding RNA
p300	Histone acetyltransferase p300
PcG	Polycomb group
PIASy	Protein inhibitor of activated STAT Y
POU2F3	POU class 2 homeobox 3
PRC	Polycomb repressive complex
QPCR	MeDIP-coupled quantitative PCR
RB	Retinoblastoma protein
RNA	Ribonucleic acid
ROCK1	Rho-associated coiled-coil containing protein kinase 1
S100P	S100 calcium binding protein P
SAHF	Senescence-associated heterochromatin foci
SAM	S-adenosylmethionine
SASP	Senescence-associated secretory phenotype
SCs	Stem cells
SP1	Specificity protein 1
SUMO	Small ubiquitin-like modifier
SUZ12	Suppressor of zeste 12
TACs	Transit-amplifying cells
TET	Ten-eleven translocation methylcytosine dioxygenases
TEWL	Trans-epidermal water loss
TLRs	Toll-like receptors
UBC9	Ubiquitin-conjugating enzyme 9
UHRF1	Ubiquitin-like with PHD and ring finger domains 1
UPS	Ubiquitin–proteasome System
UV	Ultraviolet

## References

[1] Lotfollahi Z. (2024). The Anatomy, Physiology and Function of All Skin Layers and the Impact of Ageing on the Skin. Wound Pract. Res..

[2] Dermitzakis I., Kampitsi D.D., Manthou M.E., Evangelidis P., Vakirlis E., Meditskou S., Theotokis P. (2024). Ontogeny of Skin Stem Cells and Molecular Underpinnings. Curr. Issues Mol. Biol..

[3] Abdo J.M., Sopko N.A., Milner S.M. (2020). The Applied Anatomy of Human Skin: A Model for Regeneration. Wound Med..

[4] Jiao Q., Zhi L., You B., Wang G., Wu N., Jia Y. (2024). Skin Homeostasis: Mechanism and Influencing Factors. J. Cosmet. Dermatol..

[5] Dermitzakis I., Theotokis P., Axarloglou E., Delilampou E., Manthou M.E., Meditskou S. (2024). Effects of Hazardous Chemicals on Secondary Sex Ratio: A Comprehensive Review. Chemosphere.

[6] Jo H., Brito S., Kwak B.M., Park S., Lee M.-G., Bin B.-H. (2021). Applications of Mesenchymal Stem Cells in Skin Regeneration and Rejuvenation. Int. J. Mol. Sci..

[7] Wong R., Geyer S., Weninger W., Guimberteau J.-C., Wong J.K. (2016). The Dynamic Anatomy and Patterning of Skin. Exp. Dermatol..

[8] Forslind B. (1995). The Skin: Upholder of Physiological Homeostasis. A Physiological and (Bio)Physical Study Program. Thromb. Res..

[9] Dermitzakis I., Chatzi D., Kyriakoudi S.A., Evangelidis N., Vakirlis E., Meditskou S., Theotokis P., Manthou M.E. (2024). Skin Development and Disease: A Molecular Perspective. Curr. Issues Mol. Biol..

[10] Sotiropoulou P.A., Blanpain C. (2012). Development and Homeostasis of the Skin Epidermis. Cold Spring Harb. Perspect. Biol..

[11] Greenberg E.S., Chong K.K., Huynh K.T., Tanaka R., Hoon D.S.B. (2014). Epigenetic Biomarkers in Skin Cancer. Cancer Lett..

[12] Ojeh N., Pastar I., Tomic-Canic M., Stojadinovic O. (2015). Stem Cells in Skin Regeneration, Wound Healing, and Their Clinical Applications. Int. J. Mol. Sci..

[13] Gonzales K.A.U., Fuchs E. (2017). Skin and Its Regenerative Powers: An Alliance between Stem Cells and Their Niche. Dev. Cell.

[14] Saldanha S.N., Royston K.J., Udayakumar N., Tollefsbol T.O. (2015). Epigenetic Regulation of Epidermal Stem Cell Biomarkers and Their Role in Wound Healing. Int. J. Mol. Sci..

[15] Wagner R.N., Piñón Hofbauer J., Wally V., Kofler B., Schmuth M., De Rosa L., De Luca M., Bauer J.W. (2021). Epigenetic and Metabolic Regulation of Epidermal Homeostasis. Exp. Dermatol..

[16] Blanpain C., Fuchs E. (2009). Epidermal Homeostasis: A Balancing Act of Stem Cells in the Skin. Nat. Rev. Mol. Cell Biol..

[17] Dermitzakis I., Manthou M.E., Meditskou S., Miliaras D., Kesidou E., Boziki M., Petratos S., Grigoriadis N., Theotokis P. (2022). Developmental Cues and Molecular Drivers in Myelinogenesis: Revisiting Early Life to Re-Evaluate the Integrity of CNS Myelin. Curr. Issues Mol. Biol..

[18] Dermitzakis I., Manthou M.E., Meditskou S., Tremblay M.-È., Petratos S., Zoupi L., Boziki M., Kesidou E., Simeonidou C., Theotokis P. (2023). Origin and Emergence of Microglia in the CNS—An Interesting (Hi)Story of an Eccentric Cell. Curr. Issues Mol. Biol..

[19] Dermitzakis I., Theotokis P., Evangelidis P., Delilampou E., Evangelidis N., Chatzisavvidou A., Avramidou E., Manthou M.E. (2023). CNS Border-Associated Macrophages: Ontogeny and Potential Implication in Disease. Curr. Issues Mol. Biol..

[20] Moltrasio C., Romagnuolo M., Marzano A.V. (2022). Epigenetic Mechanisms of Epidermal Differentiation. Int. J. Mol. Sci..

[21] Leśniak W. (2024). Dynamics and Epigenetics of the Epidermal Differentiation Complex. Epigenomes.

[22] Kang S., Chovatiya G., Tumbar T. (2019). Epigenetic Control in Skin Development, Homeostasis and Injury Repair. Exp. Dermatol..

[23] Perdigoto C.N., Valdes V.J., Bardot E.S., Ezhkova E. (2014). Epigenetic Regulation of Epidermal Differentiation. Cold Spring Harb. Perspect. Med..

[24] Pozzo L.D., Xu Z., Lin S., Wang J., Wang Y., Enechojo O.S., Abankwah J.K., Peng Y., Chu X., Zhou H. (2024). Role of Epigenetics in the Regulation of Skin Aging and Geroprotective Intervention: A New Sight. Biomed. Pharmacother..

[25] He J., He H., Qi Y., Yang J., Zhi L., Jia Y. (2022). Application of Epigenetics in Dermatological Research and Skin Management. J. Cosmet. Dermatol..

[26] Mulero-Navarro S., Esteller M. (2008). Epigenetic Biomarkers for Human Cancer: The Time Is Now. Crit. Rev. Oncol./Hematol..

[27] Izadi M., Sadri N., Abdi A., Serajian S., Jalalei D., Tahmasebi S. (2024). Epigenetic Biomarkers in Aging and Longevity: Current and Future Application. Life Sci..

[28] Yi J.Z., McGee J.S. (2021). Epigenetic-modifying Therapies: An Emerging Avenue for the Treatment of Inflammatory Skin Diseases. Exp. Dermatol..

[29] Orioli D., Dellambra E. (2018). Epigenetic Regulation of Skin Cells in Natural Aging and Premature Aging Diseases. Cells.

[30] Saha K., Hornyak T.J., Eckert R.L. (2013). Epigenetic Cancer Prevention Mechanisms in Skin Cancer. AAPS J..

[31] Sawada Y., Gallo R.L. (2021). Role of Epigenetics in the Regulation of Immune Functions of the Skin. J. Investig. Dermatol..

[32] Reolid A., Muñoz-Aceituno E., Abad-Santos F., Ovejero-Benito M.C., Daudén E. (2021). Epigenetics in Non-Tumor Immune-Mediated Skin Diseases. Mol. Diagn. Ther..

[33] Penta D., Somashekar B.S., Meeran S.M. (2018). Epigenetics of Skin Cancer: Interventions by Selected Bioactive Phytochemicals. Photodermatol. Photoimmunol. Photomed..

[34] Venkatesh S., Workman J.L. (2015). Histone Exchange, Chromatin Structure and the Regulation of Transcription. Nat. Rev. Mol. Cell Biol..

[35] Edwards J.R., Yarychkivska O., Boulard M., Bestor T.H. (2017). DNA Methylation and DNA Methyltransferases. Epigenetics Chromatin.

[36] Moore L.D., Le T., Fan G. (2013). DNA Methylation and Its Basic Function. Neuropsychopharmacology.

[37] Smith Z.D., Meissner A. (2013). DNA Methylation: Roles in Mammalian Development. Nat. Rev. Genet..

[38] Deaton A.M., Bird A. (2011). CpG Islands and the Regulation of Transcription. Genes Dev..

[39] Angeloni A., Bogdanovic O. (2021). Sequence Determinants, Function, and Evolution of CpG Islands. Biochem. Soc. Trans..

[40] Jeltsch A., Jurkowska R.Z. (2014). New Concepts in DNA Methylation. Trends Biochem. Sci..

[41] Lyko F. (2018). The DNA Methyltransferase Family: A Versatile Toolkit for Epigenetic Regulation. Nat. Rev. Genet..

[42] Tajima S., Suetake I., Takeshita K., Nakagawa A., Kimura H., Song J., Jeltsch A., Jurkowska R.Z. (2022). Domain Structure of the Dnmt1, Dnmt3a, and Dnmt3b DNA Methyltransferases. DNA Methyltransferases—Role and Function.

[43] Del Castillo Falconi V.M., Torres-Arciga K., Matus-Ortega G., Díaz-Chávez J., Herrera L.A. (2022). DNA Methyltransferases: From Evolution to Clinical Applications. Int. J. Mol. Sci..

[44] Richa R., Sinha R.P. (2014). Hydroxymethylation of DNA: An Epigenetic Marker. EXCLI J..

[45] Globisch D., Münzel M., Müller M., Michalakis S., Wagner M., Koch S., Brückl T., Biel M., Carell T. (2010). Tissue Distribution of 5-Hydroxymethylcytosine and Search for Active Demethylation Intermediates. PLoS ONE.

[46] Jeschke J., Collignon E., Fuks F. (2016). Portraits of TET-Mediated DNA Hydroxymethylation in Cancer. Curr. Opin. Genet. Dev..

[47] Kinney S.M., Chin H.G., Vaisvila R., Bitinaite J., Zheng Y., Estève P.-O., Feng S., Stroud H., Jacobsen S.E., Pradhan S. (2011). Tissue-Specific Distribution and Dynamic Changes of 5-Hydroxymethylcytosine in Mammalian Genomes. J. Biol. Chem..

[48] Guibert S., Weber M., Heard E. (2013). Chapter Two—Functions of DNA Methylation and Hydroxymethylation in Mammalian Development. Current Topics in Developmental Biology.

[49] Fu S., Wu H., Zhang H., Lian C.G., Lu Q. (2017). DNA Methylation/Hydroxymethylation in Melanoma. Oncotarget.

[50] Branco M.R., Ficz G., Reik W. (2011). Uncovering the Role of 5-Hydroxymethylcytosine in the Epigenome. Nat. Rev. Genet..

[51] Cheng Y., Xie N., Jin P., Wang T. (2015). DNA Methylation and Hydroxymethylation in Stem Cells. Cell Biochem. Funct..

[52] Khan S.N., Khan A.U. (2010). Role of Histone Acetylation in Cell Physiology and Diseases: An Update. Clin. Chim. Acta.

[53] Peleg S., Feller C., Ladurner A.G., Imhof A. (2016). The Metabolic Impact on Histone Acetylation and Transcription in Ageing. Trends Biochem. Sci..

[54] Seto E., Yoshida M. (2014). Erasers of Histone Acetylation: The Histone Deacetylase Enzymes. Cold Spring Harb. Perspect. Biol..

[55] Marmorstein R., Zhou M.-M. (2014). Writers and Readers of Histone Acetylation: Structure, Mechanism, and Inhibition. Cold Spring Harb. Perspect. Biol..

[56] Zhao S., Zhang X., Li H. (2018). Beyond Histone Acetylation—Writing and Erasing Histone Acylations. Curr. Opin. Struct. Biol..

[57] Ghosh K., O’Neil K., Capell B.C. (2018). Histone Modifiers: Dynamic Regulators of the Cutaneous Transcriptome. J. Dermatol. Sci..

[58] Verdone L., Caserta M., Mauro E.D. (2005). Role of Histone Acetylation in the Control of Gene Expression. Biochem. Cell Biol..

[59] Annunziato A.T., Hansen J.C. (2018). Role of Histone Acetylation in the Assembly and Modulation of Chromatin Structures. Gene Expr..

[60] Greer E.L., Shi Y. (2012). Histone Methylation: A Dynamic Mark in Health, Disease and Inheritance. Nat. Rev. Genet..

[61] Jambhekar A., Dhall A., Shi Y. (2019). Roles and Regulation of Histone Methylation in Animal Development. Nat. Rev. Mol. Cell Biol..

[62] Michalak E.M., Burr M.L., Bannister A.J., Dawson M.A. (2019). The Roles of DNA, RNA and Histone Methylation in Ageing and Cancer. Nat. Rev. Mol. Cell Biol..

[63] Cheung P., Lau P. (2005). Epigenetic Regulation by Histone Methylation and Histone Variants. Mol. Endocrinol..

[64] Di Croce L., Helin K. (2013). Transcriptional Regulation by Polycomb Group Proteins. Nat. Struct. Mol. Biol..

[65] Eckert R.L., Adhikary G., Rorke E.A., Chew Y.C., Balasubramanian S. (2011). Polycomb Group Proteins Are Key Regulators of Keratinocyte Function. J. Investig. Dermatol..

[66] Rice J.C., Allis C.D. (2001). Histone Methylation versus Histone Acetylation: New Insights into Epigenetic Regulation. Curr. Opin. Cell Biol..

[67] Lan F., Shi Y. (2009). Epigenetic Regulation: Methylation of Histone and Non-Histone Proteins. Sci. China Ser. C.

[68] Gil R.S., Vagnarelli P. (2019). Protein Phosphatases in Chromatin Structure and Function. Biochim. Et Biophys. Acta (BBA)-Mol. Cell Res..

[69] Rossetto D., Avvakumov N., Côté J. (2012). Histone Phosphorylation: A Chromatin Modification Involved in Diverse Nuclear Events. Epigenetics.

[70] Sawicka A., Seiser C. (2012). Histone H3 Phosphorylation—A Versatile Chromatin Modification for Different Occasions. Biochimie.

[71] Wirth M., Schick M., Keller U., Krönke J. (2020). Ubiquitination and Ubiquitin-Like Modifications in Multiple Myeloma: Biology and Therapy. Cancers.

[72] Pinto-Fernandez A., Kessler B.M. (2016). DUBbing Cancer: Deubiquitylating Enzymes Involved in Epigenetics, DNA Damage and the Cell Cycle As Therapeutic Targets. Front. Genet..

[73] Bach S.V., Hegde A.N. (2016). The Proteasome and Epigenetics: Zooming in on Histone Modifications. Biomol. Concepts.

[74] Jeusset L.M.-P., McManus K.J. (2019). Developing Targeted Therapies That Exploit Aberrant Histone Ubiquitination in Cancer. Cells.

[75] Oss-Ronen L., Sarusi T., Cohen I. (2022). Histone Mono-Ubiquitination in Transcriptional Regulation and Its Mark on Life: Emerging Roles in Tissue Development and Disease. Cells.

[76] Liu B., Shuai K. (2008). Regulation of the Sumoylation System in Gene Expression. Curr. Opin. Cell Biol..

[77] Shiio Y., Eisenman R.N. (2003). Histone Sumoylation Is Associated with Transcriptional Repression. Proc. Natl. Acad. Sci. USA.

[78] Nathan D., Sterner D.E., Berger S.L. (2003). Histone Modifications: Now Summoning Sumoylation. Proc. Natl. Acad. Sci. USA.

[79] Celen A.B., Sahin U. (2020). Sumoylation on Its 25th Anniversary: Mechanisms, Pathology, and Emerging Concepts. FEBS J..

[80] Ryu H.-Y., Hochstrasser M. (2021). Histone Sumoylation and Chromatin Dynamics. Nucleic Acids Res..

[81] Yao Q., Chen Y., Zhou X. (2019). The Roles of microRNAs in Epigenetic Regulation. Curr. Opin. Chem. Biol..

[82] Morales S., Monzo M., Navarro A. (2017). Epigenetic Regulation Mechanisms of microRNA Expression. Biomol. Concepts.

[83] Wang C., Wang L., Ding Y., Lu X., Zhang G., Yang J., Zheng H., Wang H., Jiang Y., Xu L. (2017). LncRNA Structural Characteristics in Epigenetic Regulation. Int. J. Mol. Sci..

[84] Lee J.T. (2012). Epigenetic Regulation by Long Noncoding RNAs. Science.

[85] Mercer T.R., Mattick J.S. (2013). Structure and Function of Long Noncoding RNAs in Epigenetic Regulation. Nat. Struct. Mol. Biol..

[86] Mattick J.S., Amaral P.P., Carninci P., Carpenter S., Chang H.Y., Chen L.-L., Chen R., Dean C., Dinger M.E., Fitzgerald K.A. (2023). Long Non-Coding RNAs: Definitions, Functions, Challenges and Recommendations. Nat. Rev. Mol. Cell Biol..

[87] Cortés-López M., Miura P. (2016). Emerging Functions of Circular RNAs. Yale J. Biol. Med..

[88] Wu J., Qi X., Liu L., Hu X., Liu J., Yang J., Yang J., Lu L., Zhang Z., Ma S. (2019). Emerging Epigenetic Regulation of Circular RNAs in Human Cancer. Mol. Ther.-Nucleic Acids.

[89] Rompolas P., Mesa K.R., Kawaguchi K., Park S., Gonzalez D., Brown S., Boucher J., Klein A.M., Greco V. (2016). Spatiotemporal Coordination of Stem Cell Commitment during Epidermal Homeostasis. Science.

[90] Gadre P., Markova P., Ebrahimkutty M., Jiang Y., Bouzada F.M., Watt F.M. (2024). Emergence and Properties of Adult Mammalian Epidermal Stem Cells. Dev. Biol..

[91] Grove G.L., Kligman A.M. (1983). Age-Associated Changes in Human Epidermal Cell Renewal1. J. Gerontol..

[92] Gonzalez-Celeiro M., Zhang B., Hsu Y.-C. (2016). Fate by Chance, Not by Choice: Epidermal Stem Cells Go Live. Cell Stem Cell.

[93] Cavalli G., Heard E. (2019). Advances in Epigenetics Link Genetics to the Environment and Disease. Nature.

[94] Lister R., Pelizzola M., Dowen R.H., Hawkins R.D., Hon G., Tonti-Filippini J., Nery J.R., Lee L., Ye Z., Ngo Q.-M. (2009). Human DNA Methylomes at Base Resolution Show Widespread Epigenomic Differences. Nature.

[95] Dauber K.L., Perdigoto C.N., Valdes V.J., Santoriello F.J., Cohen I., Ezhkova E. (2016). Dissecting the Roles of Polycomb Repressive Complex 2 Subunits in the Control of Skin Development. J. Investig. Dermatol..

[96] Bartel D.P. (2009). MicroRNAs: Target Recognition and Regulatory Functions. Cell.

[97] Bird A.P. (1980). DNA Methylation and the Frequency of CpG in Animal DNA. Nucleic Acids Res..

[98] Bestor T.H. (1992). Activation of Mammalian DNA Methyltransferase by Cleavage of a Zn Binding Regulatory Domain. EMBO J..

[99] Lei H., Oh S.P., Okano M., Jüttermann R., Goss K.A., Jaenisch R., Li E. (1996). De Novo DNA Cytosine Methyltransferase Activities in Mouse Embryonic Stem Cells. Development.

[100] Okano M., Bell D.W., Haber D.A., Li E. (1999). DNA Methyltransferases Dnmt3a and Dnmt3b Are Essential for de Novo Methylation and Mammalian Development. Cell.

[101] Sen G.L., Reuter J.A., Webster D.E., Zhu L., Khavari P.A. (2010). DNMT1 Maintains Progenitor Function in Self-Renewing Somatic Tissue. Nature.

[102] Leśniak W. (2011). Epigenetic Regulation of S100 Protein Expression. Clin. Epigenet.

[103] Andersen B., Weinberg W.C., Rennekampff O., McEvilly R.J., Bermingham J.R., Hooshmand F., Vasilyev V., Hansbrough J.F., Pittelkow M.R., Yuspa S.H. (1997). Functions of the POU Domain Genes Skn-1a/i and Tst-1/Oct-6/SCIP in Epidermal Differentiation. Genes Dev..

[104] Eckert R.L., Crish J.F., Banks E.B., Welter J.F. (1997). The Epidermis: Genes on–Genes Off. J. Investig. Dermatol..

[105] Nakamura Y., Kawachi Y., Xu X., Sakurai H., Ishii Y., Takahashi T., Otsuka F. (2007). The Combination of Ubiquitous Transcription Factors AP-1 and Sp1 Directs Keratinocyte-Specific and Differentiation-Specific Gene Expression in Vitro. Exp. Dermatol..

[106] Sharif J., Muto M., Takebayashi S., Suetake I., Iwamatsu A., Endo T.A., Shinga J., Mizutani-Koseki Y., Toyoda T., Okamura K. (2007). The SRA Protein Np95 Mediates Epigenetic Inheritance by Recruiting Dnmt1 to Methylated DNA. Nature.

[107] Bostick M., Kim J.K., Estève P.-O., Clark A., Pradhan S., Jacobsen S.E. (2007). UHRF1 Plays a Role in Maintaining DNA Methylation in Mammalian Cells. Science.

[108] Salvador J.M., Brown-Clay J.D., Fornace A.J. (2013). Gadd45 in Stress Signaling, Cell Cycle Control, and Apoptosis. Adv. Exp. Med. Biol..

[109] Barreto G., Schäfer A., Marhold J., Stach D., Swaminathan S.K., Handa V., Döderlein G., Maltry N., Wu W., Lyko F. (2007). Gadd45a Promotes Epigenetic Gene Activation by Repair-Mediated DNA Demethylation. Nature.

[110] Beck M.A., Fischer H., Grabner L.M., Groffics T., Winter M., Tangermann S., Meischel T., Zaussinger-Haas B., Wagner P., Fischer C. (2021). DNA Hypomethylation Leads to cGAS-Induced Autoinflammation in the Epidermis. EMBO J..

[111] Kawai T., Akira S. (2011). Toll-like Receptors and Their Crosstalk with Other Innate Receptors in Infection and Immunity. Immunity.

[112] Bartok E., Hartmann G. (2020). Immune Sensing Mechanisms That Discriminate Self from Altered Self and Foreign Nucleic Acids. Immunity.

[113] Li J., Jiang T.-X., Hughes M.W., Wu P., Yu J., Widelitz R.B., Fan G., Chuong C.-M. (2012). Progressive Alopecia Reveals Decreasing Stem Cell Activation Probability during Aging of Mice with Epidermal Deletion of DNA Methyltransferase 1. J. Investig. Dermatol..

[114] Rinaldi L., Avgustinova A., Martín M., Datta D., Solanas G., Prats N., Benitah S.A. (2017). Loss of Dnmt3a and Dnmt3b Does Not Affect Epidermal Homeostasis but Promotes Squamous Transformation through PPAR-γ. eLlife.

[115] Tahiliani M., Koh K.P., Shen Y., Pastor W.A., Bandukwala H., Brudno Y., Agarwal S., Iyer L.M., Liu D.R., Aravind L. (2009). Conversion of 5-Methylcytosine to 5-Hydroxymethylcytosine in Mammalian DNA by MLL Partner TET1. Science.

[116] Ito S., D’Alessio A.C., Taranova O.V., Hong K., Sowers L.C., Zhang Y. (2010). Role of Tet Proteins in 5mC to 5hmC Conversion, ES-Cell Self-Renewal and Inner Cell Mass Specification. Nature.

[117] Ito S., Shen L., Dai Q., Wu S.C., Collins L.B., Swenberg J.A., He C., Zhang Y. (2011). Tet Proteins Can Convert 5-Methylcytosine to 5-Formylcytosine and 5-Carboxylcytosine. Science.

[118] Maiti A., Drohat A.C. (2011). Thymine DNA Glycosylase Can Rapidly Excise 5-Formylcytosine and 5-Carboxylcytosine: Potential Implications for Active Demethylation of CpG Sites. J. Biol. Chem..

[119] Lu X., Zhao B.S., He C. (2015). TET Family Proteins: Oxidation Activity, Interacting Molecules, and Functions in Diseases. Chem. Rev..

[120] Li F., Yuan C.W., Xu S., Zu T., Woappi Y., Lee C.A.A., Abarzua P., Wells M., Ramsey M.R., Frank N.Y. (2020). Loss of the Epigenetic Mark 5-hmC in Psoriasis: Implications for Epidermal Stem Cell Dysregulation. J. Investig. Dermatol..

[121] Jia H.-Y., Shi Y., Luo L.-F., Jiang G., Zhou Q., Xu S.-Z., Lei T.-C. (2016). Asymmetric Stem-Cell Division Ensures Sustained Keratinocyte Hyperproliferation in Psoriatic Skin Lesions. Int. J. Mol. Med..

[122] Wang X., Liu X., Duan X., Zhu K., Zhang S., Gan L., Liu N., Jaypaul H., Makamure J.T., Ming Z. (2018). Ten-Eleven Translocation-2 Regulates DNA Hydroxymethylation Status and Psoriasiform Dermatitis Progression in Mice. Acta Derm. Venereol..

[123] Alaskhar Alhamwe B., Khalaila R., Wolf J., von Bülow V., Harb H., Alhamdan F., Hii C.S., Prescott S.L., Ferrante A., Renz H. (2018). Histone Modifications and Their Role in Epigenetics of Atopy and Allergic Diseases. Allergy Asthma Clin. Immunol..

[124] Morey L., Helin K. (2010). Polycomb Group Protein-Mediated Repression of Transcription. Trends Biochem. Sci..

[125] Kuzmichev A., Nishioka K., Erdjument-Bromage H., Tempst P., Reinberg D. (2002). Histone Methyltransferase Activity Associated with a Human Multiprotein Complex Containing the Enhancer of Zeste Protein. Genes Dev..

[126] Kirmizis A., Bartley S.M., Kuzmichev A., Margueron R., Reinberg D., Green R., Farnham P.J. (2004). Silencing of Human Polycomb Target Genes Is Associated with Methylation of Histone H3 Lys 27. Genes Dev..

[127] Luis N.M., Morey L., Mejetta S., Pascual G., Janich P., Kuebler B., Roma G., Nascimento E., Frye M., Di Croce L. (2011). Regulation of Human Epidermal Stem Cell Proliferation and Senescence Requires Polycomb- Dependent and -Independent Functions of Cbx4. Cell Stem Cell.

[128] Pivetti S., Fernandez-Perez D., D’Ambrosio A., Barbieri C.M., Manganaro D., Rossi A., Barnabei L., Zanotti M., Scelfo A., Chiacchiera F. (2019). Loss of PRC1 Activity in Different Stem Cell Compartments Activates a Common Transcriptional Program with Cell Type-Dependent Outcomes. Sci. Adv..

[129] Moritz L.E., Trievel R.C. (2018). Structure, Mechanism, and Regulation of Polycomb-Repressive Complex 2. J. Biol. Chem..

[130] Botchkarev V.A., Gdula M.R., Mardaryev A.N., Sharov A.A., Fessing M.Y. (2012). Epigenetic Regulation of Gene Expression in Keratinocytes. J. Investig. Dermatol..

[131] Ezhkova E., Pasolli H.A., Parker J.S., Stokes N., Su I., Hannon G., Tarakhovsky A., Fuchs E. (2009). Ezh2 Orchestrates Gene Expression for the Stepwise Differentiation of Tissue-Specific Stem Cells. Cell.

[132] Shen X., Kim W., Fujiwara Y., Simon M.D., Liu Y., Mysliwiec M.R., Yuan G.-C., Lee Y., Orkin S.H. (2009). Jumonji Modulates Polycomb Activity and Self-Renewal versus Differentiation of Stem Cells. Cell.

[133] Mejetta S., Morey L., Pascual G., Kuebler B., Mysliwiec M.R., Lee Y., Shiekhattar R., Di Croce L., Benitah S.A. (2011). Jarid2 Regulates Mouse Epidermal Stem Cell Activation and Differentiation. EMBO J..

[134] Sen G.L., Webster D.E., Barragan D.I., Chang H.Y., Khavari P.A. (2008). Control of Differentiation in a Self-Renewing Mammalian Tissue by the Histone Demethylase JMJD3. Genes Dev..

[135] Salminen A., Kaarniranta K., Hiltunen M., Kauppinen A. (2014). Histone Demethylase Jumonji D3 (JMJD3/KDM6B) at the Nexus of Epigenetic Regulation of Inflammation and the Aging Process. J. Mol. Med..

[136] Bartel D.P. (2004). MicroRNAs: Genomics, Biogenesis, Mechanism, and Function. Cell.

[137] Yi R., Poy M.N., Stoffel M., Fuchs E. (2008). A Skin microRNA Promotes Differentiation by Repressing “Stemness”. Nature.

[138] Lena A.M., Shalom-Feuerstein R., Rivetti di Val Cervo P., Aberdam D., Knight R.A., Melino G., Candi E. (2008). miR-203 Represses “stemness” by Repressing DeltaNp63. Cell Death Differ..

[139] Kai-Hong J., Jun X., Kai-Meng H., Ying W., Hou-Qi L. (2007). P63 Expression Pattern during Rat Epidermis Morphogenesis and the Role of P63 as a Marker for Epidermal Stem Cells. J. Cutan. Pathol..

[140] The Role of P63 in Development and Differentiation of the Epidermis—PubMed. https://pubmed.ncbi.nlm.nih.gov/14757276/.

[141] Parsa R., Yang A., McKeon F., Green H. (1999). Association of P63 with Proliferative Potential in Normal and Neoplastic Human Keratinocytes. J. Investig. Dermatol..

[142] Le Provost G.S., Debret R., Cenizo V., Aimond G., Pez F., Kaniewski B., André V., Sommer P. (2010). Lysyl Oxidase Silencing Impairs Keratinocyte Differentiation in a Reconstructed-Epidermis Model. Exp. Dermatol..

[143] Boufraqech M., Nilubol N., Zhang L., Gara S.K., Sadowski S.M., Mehta A., He M., Davis S., Dreiling J., Copland J.A. (2015). miR30a Inhibits LOX Expression and Anaplastic Thyroid Cancer Progression. Cancer Res..

[144] Muther C., Jobeili L., Garion M., Heraud S., Thepot A., Damour O., Lamartine J. (2017). An Expression Screen for Aged-Dependent microRNAs Identifies miR-30a as a Key Regulator of Aging Features in Human Epidermis. Aging.

[145] Pickup M.E., Hu A., Patel H.J., Ahmed M.I. (2023). MicroRNA-148a Controls Epidermal and Hair Follicle Stem/Progenitor Cells by Modulating the Activities of ROCK1 and ELF5. J. Investig. Dermatol..

[146] Sieck G.C. (2018). Physiology in Perspective: Understanding the Aging Process. Physiology.

[147] Rando T.A. (2006). Stem Cells, Ageing and the Quest for Immortality. Nature.

[148] Gorgoulis V., Adams P.D., Alimonti A., Bennett D.C., Bischof O., Bishop C., Campisi J., Collado M., Evangelou K., Ferbeyre G. (2019). Cellular Senescence: Defining a Path Forward. Cell.

[149] Zampetidis C.P., Galanos P., Angelopoulou A., Zhu Y., Polyzou A., Karamitros T., Kotsinas A., Lagopati N., Mourkioti I., Mirzazadeh R. (2021). A Recurrent Chromosomal Inversion Suffices for Driving Escape from Oncogene-Induced Senescence via subTAD Reorganization. Mol. Cell.

[150] Rattan S.I.S. (2006). Theories of Biological Aging: Genes, Proteins, and Free Radicals. Free Radic. Res..

[151] Yaar M., Gilchrest B.A. (2007). Photoageing: Mechanism, Prevention and Therapy. Br. J. Dermatol..

[152] Krajčovičová-Kudláčková M., Valachovičová M., Pauková V., Dušinská M. (2008). Effects of Diet and Age on Oxidative Damage Products in Healthy Subjects. Physiol. Res..

[153] Zouboulis C.C., Chen W.-C., Thornton M.J., Qin K., Rosenfield R. (2007). Sexual Hormones in Human Skin. Horm. Metab. Res..

[154] Targeting Multiple Hallmarks of Skin Aging: Preclinical and Clinical Efficacy of a Novel Growth Factor-Based Skin Care Serum—PubMed. https://pubmed.ncbi.nlm.nih.gov/36374431/.

[155] Inflammation, Epigenetics, and Metabolism Converge to Cell Senescence and Ageing: The Regulation and Intervention—PubMed. https://pubmed.ncbi.nlm.nih.gov/34176928/.

[156] López-Otín C., Blasco M.A., Partridge L., Serrano M., Kroemer G. (2013). The Hallmarks of Aging. Cell.

[157] López-Otín C., Blasco M.A., Partridge L., Serrano M., Kroemer G. (2023). Hallmarks of Aging: An Expanding Universe. Cell.

[158] Lavker R.M., Zheng P.S., Dong G. (1987). Aged Skin: A Study by Light, Transmission Electron, and Scanning Electron Microscopy. J. Investig. Dermatol..

[159] Matsumura H., Mohri Y., Binh N.T., Morinaga H., Fukuda M., Ito M., Kurata S., Hoeijmakers J., Nishimura E.K. (2016). Hair Follicle Aging Is Driven by Transepidermal Elimination of Stem Cells via COL17A1 Proteolysis. Science.

[160] Nishimura E.K., Granter S.R., Fisher D.E. (2005). Mechanisms of Hair Graying: Incomplete Melanocyte Stem Cell Maintenance in the Niche. Science.

[161] Lowry W.E. (2020). Its Written All over Your Face: The Molecular and Physiological Consequences of Aging Skin. Mech. Ageing Dev..

[162] Puizina-Ivić N. (2008). Skin Aging. Acta Dermatovenerol. Alp. Pannonica Adriat..

[163] Braga D.L., Mousovich-Neto F., Tonon-da-Silva G., Salgueiro W.G., Mori M.A. (2020). Epigenetic Changes during Ageing and Their Underlying Mechanisms. Biogerontology.

[164] Andersen B., Millar S. (2021). Skin Epigenetics. Exp. Dermatol..

[165] Wlaschek M., Maity P., Makrantonaki E., Scharffetter-Kochanek K. (2021). Connective Tissue and Fibroblast Senescence in Skin Aging. J. Investig. Dermatol..

[166] Talbott H.E., Mascharak S., Griffin M., Wan D.C., Longaker M.T. (2022). Wound Healing, Fibroblast Heterogeneity, and Fibrosis. Cell Stem Cell.

[167] Tigges J., Krutmann J., Fritsche E., Haendeler J., Schaal H., Fischer J.W., Kalfalah F., Reinke H., Reifenberger G., Stühler K. (2014). The Hallmarks of Fibroblast Ageing. Mech. Ageing Dev..

[168] Calleja-Agius J., Brincat M., Borg M. (2013). Skin Connective Tissue and Ageing. Best Pract. Res. Clin. Obstet. Gynaecol..

[169] Ishimi Y., Kojima M., Takeuchi F., Miyamoto T., Yamada M., Hanaoka F. (1987). Changes in Chromatin Structure during Aging of Human Skin Fibroblasts. Exp. Cell Res..

[170] Plikus M.V., Wang X., Sinha S., Forte E., Thompson S.M., Herzog E.L., Driskell R.R., Rosenthal N., Biernaskie J., Horsley V. (2021). Fibroblasts: Origins, Definitions, and Functions in Health and Disease. Cell.

[171] Narita M., Nũnez S., Heard E., Narita M., Lin A.W., Hearn S.A., Spector D.L., Hannon G.J., Lowe S.W. (2003). Rb-Mediated Heterochromatin Formation and Silencing of E2F Target Genes during Cellular Senescence. Cell.

[172] Ferbeyre G., de Stanchina E., Lin A.W., Querido E., McCurrach M.E., Hannon G.J., Lowe S.W. (2002). Oncogenic Ras and P53 Cooperate to Induce Cellular Senescence. Mol. Cell Biol..

[173] Wang B., Du R., Xiao X., Deng Z.-L., Jian D., Xie H.-F., Li J. (2017). Microrna-217 Modulates Human Skin Fibroblast Senescence by Directly Targeting DNA Methyltransferase 1. Oncotarget.

[174] Svedružić Ž.M. (2011). Dnmt1 Structure and Function. Prog. Mol. Biol. Transl. Sci..

[175] De Paoli-Iseppi R., Deagle B.E., McMahon C.R., Hindell M.A., Dickinson J.L., Jarman S.N. (2017). Measuring Animal Age with DNA Methylation: From Humans to Wild Animals. Front. Genet..

[176] Fisher G.J., Varani J., Voorhees J.J. (2008). Looking Older: Fibroblast Collapse and Therapeutic Implications. Arch. Dermatol..

[177] Varani J., Dame M.K., Rittie L., Fligiel S.E.G., Kang S., Fisher G.J., Voorhees J.J. (2006). Decreased Collagen Production in Chronologically Aged Skin: Roles of Age-Dependent Alteration in Fibroblast Function and Defective Mechanical Stimulation. Am. J. Pathol..

[178] Xie H.-F., Liu Y.-Z., Du R., Wang B., Chen M.-T., Zhang Y.-Y., Deng Z.-L., Li J. (2017). miR-377 Induces Senescence in Human Skin Fibroblasts by Targeting DNA Methyltransferase 1. Cell Death Dis..

[179] Ciccarone F., Malavolta M., Calabrese R., Guastafierro T., Bacalini M.G., Reale A., Franceschi C., Capri M., Hervonen A., Hurme M. (2016). Age-Dependent Expression of 1 and 3B in PBMCs from a Large European Population Enrolled in the MARK-AGE Study. Aging Cell.

[180] Qian H., Xu X. (2014). Reduction in DNA Methyltransferases and Alteration of DNA Methylation Pattern Associate with Mouse Skin Ageing. Exp. Dermatol..

[181] Moulin L., Cenizo V., Antu A.N., André V., Pain S., Sommer P., Debret R. (2017). Methylation of LOXL1 Promoter by DNMT3A in Aged Human Skin Fibroblasts. Rejuvenation Res..

[182] Wagenseil J.E., Mecham R.P. (2007). New Insights into Elastic Fiber Assembly. Birth Defects Res. C Embryo Today.

[183] Liu X., Zhao Y., Gao J., Pawlyk B., Starcher B., Spencer J.A., Yanagisawa H., Zuo J., Li T. (2004). Elastic Fiber Homeostasis Requires Lysyl Oxidase-like 1 Protein. Nat. Genet..

[184] Xiao Y., Word B., Starlard-Davenport A., Haefele A., Lyn-Cook B.D., Hammons G. (2008). Age and Gender Affect DNMT3a and DNMT3b Expression in Human Liver. Cell Biol. Toxicol..

[185] Azzi A., Dallmann R., Casserly A., Rehrauer H., Patrignani A., Maier B., Kramer A., Brown S.A. (2014). Circadian Behavior Is Light-Reprogrammed by Plastic DNA Methylation. Nat. Neurosci..

[186] Morgan A.E., Davies T.J., Mc Auley M.T. (2018). The Role of DNA Methylation in Ageing and Cancer. Proc. Nutr. Soc..

[187] Mc Auley M.T. (2021). DNA Methylation in Genes Associated with the Evolution of Ageing and Disease: A Critical Review. Ageing Res. Rev..

[188] Fraga M.F., Esteller M. (2007). Epigenetics and Aging: The Targets and the Marks. Trends Genet..

[189] Terlecki-Zaniewicz L., Pils V., Bobbili M.R., Lämmermann I., Perrotta I., Grillenberger T., Schwestka J., Weiß K., Pum D., Arcalis E. (2019). Extracellular Vesicles in Human Skin: Cross-Talk from Senescent Fibroblasts to Keratinocytes by miRNAs. J. Investig. Dermatol..

[190] Focus on Extracellular Vesicles: Physiological Role and Signalling Properties of Extracellular Membrane Vesicles—PubMed. https://pubmed.ncbi.nlm.nih.gov/26861302/.

[191] Cicero A.L., Delevoye C., Gilles-Marsens F., Loew D., Dingli F., Guéré C., André N., Vié K., van Niel G., Raposo G. (2015). Exosomes Released by Keratinocytes Modulate Melanocyte Pigmentation. Nat. Commun..

[192] Huang P., Bi J., Owen G.R., Chen W., Rokka A., Koivisto L., Heino J., Häkkinen L., Larjava H. (2015). Keratinocyte Microvesicles Regulate the Expression of Multiple Genes in Dermal Fibroblasts. J. Investig. Dermatol..

[193] Merjaneh M., Langlois A., Larochelle S., Cloutier C.B., Ricard-Blum S., Moulin V.J. (2017). Pro-Angiogenic Capacities of Microvesicles Produced by Skin Wound Myofibroblasts. Angiogenesis.

[194] Zhou L., Wang H., Jing J., Yu L., Wu X., Lu Z. (2018). Regulation of Hair Follicle Development by Exosomes Derived from Dermal Papilla Cells. Biochem. Biophys. Res. Commun..

[195] Lopes-Paciencia S., Saint-Germain E., Rowell M.-C., Ruiz A.F., Kalegari P., Ferbeyre G. (2019). The Senescence-Associated Secretory Phenotype and Its Regulation. Cytokine.

[196] Terlecki-Zaniewicz L., Lämmermann I., Latreille J., Bobbili M.R., Pils V., Schosserer M., Weinmüllner R., Dellago H., Skalicky S., Pum D. (2018). Small Extracellular Vesicles and Their miRNA Cargo Are Anti-Apoptotic Members of the Senescence-Associated Secretory Phenotype. Aging.

[197] Röck K., Tigges J., Sass S., Schütze A., Florea A.-M., Fender A.C., Theis F.J., Krutmann J., Boege F., Fritsche E. (2015). miR-23a-3p Causes Cellular Senescence by Targeting Hyaluronan Synthase 2: Possible Implication for Skin Aging. J. Investig. Dermatol..

[198] Tzellos T.G., Klagas I., Vahtsevanos K., Triaridis S., Printza A., Kyrgidis A., Karakiulakis G., Zouboulis C.C., Papakonstantinou E. (2009). Extrinsic Ageing in the Human Skin Is Associated with Alterations in the Expression of Hyaluronic Acid and Its Metabolizing Enzymes. Exp. Dermatol..

[199] Rivetti di Val Cervo P., Lena A.M., Nicoloso M., Rossi S., Mancini M., Zhou H., Saintigny G., Dellambra E., Odorisio T., Mahé C. (2012). P63-microRNA Feedback in Keratinocyte Senescence. Proc. Natl. Acad. Sci. USA.

[200] Mancini M., Saintigny G., Mahé C., Annicchiarico-Petruzzelli M., Melino G., Candi E. (2012). MicroRNA-152 and -181a Participate in Human Dermal Fibroblasts Senescence Acting on Cell Adhesion and Remodeling of the Extra-Cellular Matrix. Aging.

[201] Wang Y., Scheiber M.N., Neumann C., Calin G.A., Zhou D. (2011). MicroRNA Regulation of Ionizing Radiation-Induced Premature Senescence. Int. J. Radiat. Oncol. Biol. Phys..

[202] Gerasymchuk M., Cherkasova V., Kovalchuk O., Kovalchuk I. (2020). The Role of microRNAs in Organismal and Skin Aging. Int. J. Mol. Sci..

[203] Li W., Zhou B.-R., Hua L.-J., Guo Z., Luo D. (2013). Differential miRNA Profile on Photoaged Primary Human Fibroblasts Irradiated with Ultraviolet A. Tumour Biol..

[204] Krutmann J. (2001). The Role of UVA Rays in Skin Aging. Eur. J. Dermatol..

[205] Bauwens E., Parée T., Meurant S., Bouriez I., Hannart C., Wéra A.-C., Khelfi A., Fattaccioli A., Burteau S., Demazy C. (2023). Senescence Induced by UVB in Keratinocytes Impairs Amino Acids Balance. J. Investig. Dermatol..

[206] Rübe C.E., Bäumert C., Schuler N., Isermann A., Schmal Z., Glanemann M., Mann C., Scherthan H. (2021). Human Skin Aging Is Associated with Increased Expression of the Histone Variant H2A.J in the Epidermis. NPJ Aging Mech. Dis..

[207] Salminen A., Kaarniranta K., Kauppinen A. (2022). Photoaging: UV Radiation-Induced Inflammation and Immunosuppression Accelerate the Aging Process in the Skin. Inflamm. Res..

[208] Mancini M., Lena A.M., Saintigny G., Mahé C., Di Daniele N., Melino G., Candi E. (2014). MicroRNAs in Human Skin Ageing. Ageing Res. Rev..

[209] Satoh A., Brace C.S., Rensing N., Cliften P., Wozniak D.F., Herzog E.D., Yamada K.A., Imai S.-I. (2013). Sirt1 Extends Life Span and Delays Aging in Mice through the Regulation of Nk2 Homeobox 1 in the DMH and LH. Cell Metab..

[210] Herranz D., Cañamero M., Mulero F., Martinez-Pastor B., Fernandez-Capetillo O., Serrano M. (2010). Sirt1 Improves Healthy Ageing and Protects from Metabolic Syndrome-Associated Cancer Syndrome. Nat. Commun..

[211] Wang Y., Yuan Q., Xie L. (2018). Histone Modifications in Aging: The Underlying Mechanisms and Implications. Curr. Stem Cell Res. Ther..

[212] McCauley B.S., Dang W. (2014). Histone Methylation and Aging: Lessons Learned from Model Systems. Biochim. Biophys. Acta.

[213] Koester J., Miroshnikova Y.A., Ghatak S., Chacón-Martínez C.A., Morgner J., Li X., Atanassov I., Altmüller J., Birk D.E., Koch M. (2021). Niche Stiffening Compromises Hair Follicle Stem Cell Potential during Ageing by Reducing Bivalent Promoter Accessibility. Nat. Cell Biol..

[214] Lee J., Kang S., Lilja K.C., Colletier K.J., Scheitz C.J.F., Zhang Y.V., Tumbar T. (2016). Signalling Couples Hair Follicle Stem Cell Quiescence with Reduced Histone H3 K4/K9/K27me3 for Proper Tissue Homeostasis. Nat. Commun..

